# Programmed Cell Death-Ligand 1 Expression and Clinical Outcomes Among Patients with Resected, Early-Stage Non-Small Cell Lung Cancer: A Real-World Study

**DOI:** 10.3390/curroncol31110497

**Published:** 2024-10-31

**Authors:** Parneet K. Cheema, Iqra Syed, Femida Gwadry-Sridhar, Muhammad Rakibuz-Zaman, Robin Sachdeva, Alec Pencz, Luna Zhan, Katrina Hueniken, Devalben Patel, Karmugi Balaratnam, Khaleeq Khan, Benjamin Grant, Brandon S. Sheffield, M. Elizabeth O. Locke, Daniel Moldaver, Mary Kate Shanahan, Geoffrey Liu, M. Sara Kuruvilla

**Affiliations:** 1 William Osler Health System, University of Toronto, 2100 Bovaird Drive, Brampton, ON L6R 3J7, Canada; 2 Department of Medicine, University of Toronto, 27 King’s College Circle, Toronto, ON M5S 1A1, Canada; 3 AstraZeneca Canada, Inc., 5000-1004 Middlegate Road, Mississauga, ON L4Y 1M4, Canadamoldaverd@gmail.com (D.M.);; 4 Pulse Infoframe Inc., 235 N Centre Road #101, London, ON N5X 4E7, Canada; 5 Verspeeten Family Cancer Center and Lawson Health Research Institute, London Health Sciences Center, London, ON N6A 5W9, Canada; 6 Princess Margaret Cancer Centre, University Health Network, 610 University Avenue, Toronto, ON M5G 2M9, Canada; 7 Department of Oncology, Schulich School of Medicine and Dentistry, Western University, 1151 Richmond St, London, ON N6A 3K7, Canada

**Keywords:** non-small cell lung cancer, early stage, real world, programmed cell death-ligand 1, prevalence, recurrence, survival

## Abstract

Treatment options for non-small cell lung cancer (NSCLC) are evolving, given recent and expected approvals of immune checkpoint inhibitors (ICIs) targeting programmed cell death-(ligand) 1 (PD-1/PD-L1). We retrospectively evaluated outcomes among patients with resected stage IB-IIIA NSCLC tumors expressing PD-L1 using PALEOS (Pan-cAnadian Lung cancEr Observational Study) data (2016–2019). Key outcomes included PD-L1 expression rate and treatment patterns, recurrence, and median overall (mOS) and disease-free survival (mDFS) among PD-L1+ patients. Among 539 PD-L1–tested patients, 317 (58.8%) were PD-L1+ (≥1%). At diagnosis, 35.3%, 39.8%, and 24.9% of PD-L1+ patients had stage IB, II, or IIIA disease. Forty-one percent had received adjuvant therapy. At 22.6 months (median follow-up), first disease recurrence had occurred in 31.9% of patients, primarily at metastatic sites. After first metastatic recurrence, ICI regimens were the most common first systemic therapy (29.8%). mOS was not reached; mDFS was 40.0 months. At four years, DFS probability was 44%. Four-year OS and DFS rates were generally similar when stratified by PD-L1 expression (1–49% vs. ≥50%). These findings underscore the generally poor outcomes experienced by patients with early-stage, resected, PD-L1+ NSCLC after treatment with available adjuvant therapies, and provide context to recent and emerging trials of new treatment options.

## 1. Introduction

In Canada and worldwide, lung cancer remains a leading cause of cancer incidence and death among both men and women [1,2]. The most common disease subtype is non-small cell lung cancer (NSCLC), which is estimated to account for 88% of all lung cancer cases in Canada [3]. At diagnosis, approximately half of patients with NSCLC have stage I-III disease [3] and about one-third are eligible for surgical resection [4,5], the preferred treatment option for most individuals with stage IB-IIIA disease [6,7,8,9]. In an effort to eradicate residual NSCLC post-surgery and minimize the risk of disease recurrence [10], adjuvant chemotherapy has historically been recommended for suitable patients [6,7,8,9], as such treatment can prolong overall survival (OS) and disease-free survival (DFS) among patients with resected, early-stage disease [7,11,12]. However, recurrence remains a concern after adjuvant chemotherapy [13,14]: at five years, recurrence rates are approximately 45% in stage IB, 62% in stage II, and 76% in stage III disease [11]. Comparable findings have been observed with use of neoadjuvant chemotherapy [15].

The inordinately high rates of recurrence observed with (neo)adjuvant chemotherapy in early-stage NSCLC have prompted the evaluation of existing and new cancer therapies in adjuvant, neoadjuvant, and perioperative (i.e., neoadjuvant followed by adjuvant) settings [10,16,17]. Several of these options, such as the third-generation epidermal growth factor receptor tyrosine kinase inhibitor (EGFR-TKI) osimertinib and several programmed cell death-1 receptor/programmed cell death-ligand 1 inhibitors (PD-1/PD-L1), are already well-established in the management of advanced NSCLC [8]. Based on the results of the ADAURA trial [18], adjuvant use of osimertinib has become part of the recommended standard of care for patients with resected stage IB-IIIA (American Joint Committee on Cancer [AJCC] 7th edition) NSCLC that harbors sensitizing *EGFR* mutations (*EGFR*m) [8,19,20]. Similarly, results of phase III trials of atezolizumab (a PD-L1 inhibitor) and pembrolizumab (a PD-1 inhibitor) monotherapy [21,22] and nivolumab (PD-1 inhibitor) used in combination with chemotherapy [23] have led to the approval of these agents in adjuvant or neoadjuvant settings, with specific indications varying worldwide. Additionally, pembrolizumab, durvalumab (a PD-L1 inhibitor), and nivolumab have been associated with positive results in the perioperative NSCLC setting of the KEYNOTE-671, AEGEAN, and CheckMate 77T trials, respectively, with KEYNOTE-671 recently showing an OS benefit [24,25,26,27]. Other immune checkpoint inhibitor (ICI) therapies are currently under evaluation for use in early-stage NSCLC, with numerous phase III clinical trial readouts expected over the next several years.

Given the recent approval of ICIs in early-stage settings and impending clinical trial results, it is of interest to understand the real-world characteristics, treatment, and clinical outcomes of patients with early-stage resected NSCLC who may be eligible for these therapies. There is a paucity of published information for this population in general and in particular for those with expression of PD-L1. Therefore, the goal of this study was to identify the prevalence of PD-L1 expression (≥1%) among patients with resected stage IB-IIIA NSCLC receiving treatment in Ontario, Canada. Additionally, patient demographics, clinical characteristics, treatment patterns, and recurrence and survival outcomes were analyzed among patients with PD-L1-positive disease (expression ≥ 1%).

## 2. Materials and Methods

### 2.1. Study Design and Patient Eligibility Criteria

A retrospective, longitudinal, observational, cohort study was conducted to analyze outcomes among surgically resected patients with early-stage NSCLC. Included patients were adults aged ≥ 18 years diagnosed with stage IB-IIIA disease (AJCC 7th edition) tested for PD-L1 expression between 2016 and 2019. All patients had received surgical resection ± adjuvant therapy for NSCLC. Patients who had received neoadjuvant therapy were excluded from the study.

### 2.2. Data Source and Study Timeframe

Data were derived from the Pan-cAnadian Lung cancEr Observational Study (PALEOS), a multicenter, retrospective evaluation of patients with early-stage NSCLC. The data were captured from three large cancer centers located in Ontario, Canada, which offer PD-L1 (non-squamous and squamous histologies) and *EGFR*m testing (non-squamous) to all patients with NSCLC at the time of diagnosis: the London Health Sciences Centre (LHSC), Princess Margaret Cancer Centre (PM), and William Osler Health System (WOHS). All information was collected on the Pulse Infoframe platform, which is mapped to the Observational Medical Outcomes Partnership (OMOP) data standard. The study period spanned 2016 through 2019, as PD-L1 testing was initiated at all centers in 2016. Reflex testing for PD-L1 at the time of diagnosis of non-squamous and squamous histology was initiated in late 2017 at LHSC and WOHS and in early 2018 at PM. Reflex testing for *EGFR*m has been conducted since 2016 at all three centers for patients with NSCLC of non-squamous cell carcinoma histology. All testing laboratories employed PD-L1 and *EGFR* assays validated for use in NSCLC. At the time of the study, all laboratories participated in regular external proficiency testing, were fully accredited, and were in good standing with local practices. All patients who fulfilled the study eligibility criteria and were seen at LHSC, PM, or WOHS were recruited, thereby limiting the possibility of bias.

### 2.3. Study Outcomes

Study outcomes included patient demographics and clinical characteristics, PD-L1 testing data, and prevalence rates for PD-L1 expression (≥1%) and *EGFR*m co-expression. Among patients with PD-L1+ NSCLC, the following outcomes were also evaluated: demographics and clinical characteristics, treatment patterns (i.e., reception of adjuvant therapy and treatment type after first metastatic recurrence), rates of first locoregional and metastatic recurrence, and OS and DFS. Patient race was categorized according to self-identification and physician reporting; these data were only available from WOHS and PM, not LHSC. Overall survival was defined as the time from surgical resection until death; patients without death events had OS censored at the last date they were known to be alive. Disease-free survival was defined as the length of time from the index date (i.e., resection) to the date of a recurrence event (as determined by the treating clinician based on chart review) or death; patients without recurrence or death events had DFS censored at their last clinic visit.

### 2.4. Data Analysis

PD-L1 prevalence was calculated as the proportion of PD-L1+ patients among all patients who underwent PD-L1 testing. Demographics, clinical characteristics, treatment patterns, disease recurrence, and survival outcomes were evaluated for the PD-L1+ cohort, with stratification based on disease stage and other characteristics of interest (e.g., PD-L1 expression 1–49% or ≥50%; *EGFR*m co-expression). Continuous study outcomes were reported descriptively using mean and standard deviation (SD), while categorical study outcomes were reported using frequencies and percentages and included 95% confidence intervals (CIs) for key outcome variables. Kaplan–Meier curves for OS and DFS were estimated overall and stratified across subgroups of interest (e.g., disease stage, treatment, *EGFR*m co-expression), with medians and two- and four-year rates reported. Unadjusted differences in OS and DFS by disease stage, PD-L1 expression, and *EGFR*m status were tested using the log-rank test, as visual inspection of the curves did not reveal meaningful deviations in proportional hazards. No other statistical testing was performed in this descriptive study.

### 2.5. Ethics

The study was approved by the Ontario Cancer Research Ethics Board (OCREB), a centralized Research Ethics Board (REB) for oncology studies in Ontario.

## 3. Results

### 3.1. PD-L1 Prevalence Cohort

A total of 539 patients with surgically resected, early-stage NSCLC underwent testing for PD-L1 expression between 2016 and 2019 at LHSC, PM, and WOHS. The rate of PD-L1 expression ≥1% was 58.8% (n = 317), with 25.4% of all tested patients having expression ≥50% (43.2% of PD-L1+ patients) (Table 1). Compared with PD-L1–negative patients, numerically more PD-L1+ patients were current or former smokers and had squamous cell histology. Among individuals with known *EGFR*m status (n = 390; 221 PD-L1+ and 169 PD-L1–negative patients), fewer PD-L1+ than PD-L1–negative patients were also *EGFR*m-positive (36 (16.3%) vs. 46 (27.2%), respectively; Table 1).

### 3.2. PD-L1+ Cohort

#### 3.2.1. Patient Demographics and Clinical Characteristics

In the PD-L1+ (≥1%) cohort (n = 317), the mean (SD) age at diagnosis was 70.2 (8.8) years (Table 2). Two hundred and forty-one patients (76.0%) were aged ≥65 years and females and males were similarly represented (51.1% and 48.9%, respectively). Most patients had stage IB (35.3%) or stage II (39.8%) NSCLC at diagnosis, were current or former smokers (85.2%), and had adenocarcinoma histology (62.5%). Almost all patients (95.9%) had no macroscopic or microscopic residual tumor (R0) after surgical resection ± adjuvant therapy. Patient characteristics were also evaluated among PD-L1+, *EGFR*m-negative (wild type) patients (see Supplementary Materials, Table S1).

#### 3.2.2. Disease Recurrence

At a median follow-up of 22.6 months, about one-third of PD-L1+ patients (n = 101; 31.9%) had experienced a first disease recurrence (see Supplementary Materials, Table S2). Among those experiencing such recurrence, a larger proportion had received surgical resection + adjuvant therapy than resection alone (46.6% vs. 21.5%, respectively) and recurred with metastatic rather than locoregional disease (70.3% vs 29.7%, respectively). After the lymph nodes (36 (27.9%)), the lung and central nervous system (CNS) were the most frequent sites of first disease recurrence (25 (19.4%) and 19 (14.7%) of 129 sites, respectively; see Supplementary Materials, Table S3).

Among PD-L1+ patients with known *EGFR*m status, first disease recurrence (locoregional or metastatic) occurred in a higher proportion of *EGFR*m-positive patients than *EGFR*m-negative patients (19/36 (52.8%) vs. 69/185 (37.3%)). Among the *EGFR*m-positive patients, first recurrence occurred most frequently in the lung (7/19 (36.8%); among the *EGFR*m-negative patients, first recurrence was most frequent in the CNS (15/69 (21.7%)).

#### 3.2.3. Treatment Patterns

A higher proportion of PD-L1+ patients underwent surgical resection alone (58.7%, of whom 80.1% were aged ≥65 years) than surgical resection + adjuvant therapy (41.3% (with 70.2% ≥65 years of age)) (Table 2). This finding was driven largely by patients with stage IB disease, who represented 54.8% (102/186) of patients undergoing resection alone—only 8.9% (10/112) of all stage IB patients had resection followed by adjuvant therapy. The majority of patients with stage II or IIIA disease had received surgical resection and adjuvant therapy (68/126 (54.0%) and 53/79 (67.1%), respectively). A smaller proportion of PD-L1+/*EGFR*m-negative patients received resection + adjuvant therapy (75/185 (40.5%)) than PD-L1+/*EGFR*m-positive patients (21/36 (58.3%)) (Table 2); however, these results were not available by disease stage.

Among the 131 PD-L1+ patients who received adjuvant therapy, chemotherapy alone was the most frequently used systemic treatment (78.6% of patients) (see Supplementary Materials, Table S4). A minority of the adjuvant-treated patients received chemotherapy + RT (3.8%), chemotherapy + ICI only (1.5%), or chemotherapy + ICI + RT (1.5%); ICI therapy was received through a clinical trial. Eighteen patients (13.7%) received adjuvant RT alone. Treatment patterns were similar among patients with PD-L1+/*EGFR*m-positive disease, with chemotherapy alone being the most commonly received adjuvant therapy (15/21 patients (71.4%); see Supplementary Materials, Table S5).

Among PD-L1+ patients with data available for the first treatment received after first metastatic recurrence, ICI combinations were the most frequently received systemic regimen (17/57 patients; 29.8%; see Supplementary Materials, Table S6). Radiation therapy alone was received by 16 (28.1%) of patients, while 8 (14.0%) did not receive therapy in the metastatic setting. Among patients with *EGFR*m-positive disease, tyrosine kinase inhibitors (7/10 patients; 70%) were the most frequently received systemic therapies in the metastatic setting.

#### 3.2.4. Overall and Disease-Free Survival

**Figure 1:**
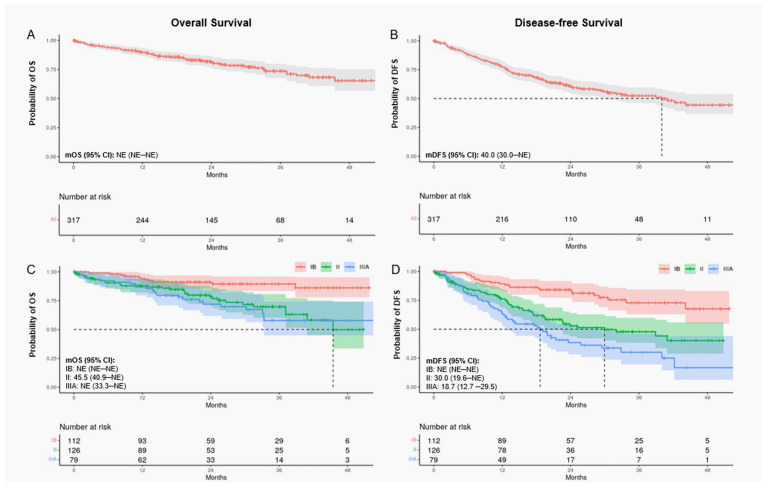
Kaplan–Meier curves for OS and DFS among PD-L1+ patients. All patients (**A**,**B**) and stratified by disease stage at diagnosis (**C**,**D**). Shaded areas represent 95% CIs. Curves C and D show statistically significant separation in time to death between stage IB, II, and IIIA patients (log-rank *p*-value: < 0.001). CI, confidence interval; DFS, disease-free survival; NE, not estimable; OS, overall survival.

**Figure 2:**
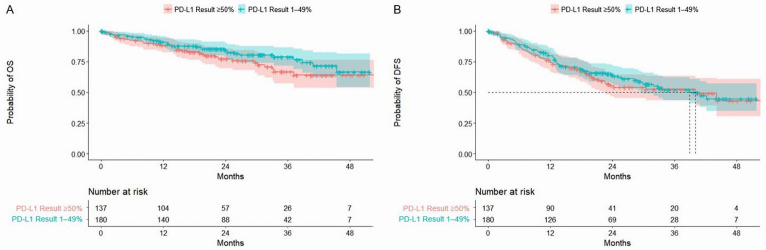
Kaplan–Meier curves for OS (**A**) and DFS (**B**) stratified by PD-L1 expression 1%-49% and ≥50%. Shaded areas represent 95% CIs. In both comparisons, there was no statistically significant difference in the time to death between patients with PD-L1 expression 1–49% versus ≥50% (log-rank *p*-value: > 0.05). CI, confidence interval; DFS, disease-free survival; PD-L1, programmed cell death-ligand 1.

Median follow-up was 24.4 months for patients with stage IB disease and 20.6 months for those with stage II or IIIA disease. In the overall cohort and across most patient subgroups, mOS was not reached (Table 3; Figure 1). At two years, the probability of OS was 81% (95% CI: 77–86%) for all PD-L1+ patients and declined with advancing disease stage at diagnosis (stage IB: 91%, 95% CI: 86–97%; stage II: 78%, 95% CI: 70–87%; stage IIIA: 72%, 95% CI: 62–84%; see Supplementary Materials, Table S7). The probability of OS did not differ significantly between patients with PD-L1 expression 1–49% versus ≥50% (Figure 2) and was generally comparable across other subgroups (see Supplementary Materials, Table S7, and Figure S1). At four years, the probability of survival was 65% (95% CI: 57–75%) among all patients, 86% (95% CI: 78–95%) among stage IB patients, 50% (95% CI: 34–74%) among stage II patients, and 58% (95% CI: 45–74%) among stage IIIA patients. Compared with patients diagnosed with stage IB disease, those diagnosed with stage II or IIIA disease had a higher risk of death within four years (stage II: hazard ratio (HR) 3.01, 95% CI: 1.51–6.00; stage IIIA: HR 3.52, 95% CI: 1.72–7.19). Patients who underwent resection alone had a numerically higher probability of survival at four years than those who also received adjuvant therapy (74% (95% CI: 66–84%) vs. 56% (95% CI: 42–75%)), though rates were similar at two years (82% (95% CI: 76–88%) and 81% (74–88%)). Results for OS were similar across other subgroups.

In the PD-L1+ population, mDFS was 40.0 months (95% CI: 30.0–not estimable (NE)) and declined with advancing disease stage, from NE in stage IB to 30.0 months (95% CI: 19.6–NE) in stage II and 18.7 months (95% CI: 12.7–29.5) in stage IIIA (Table 3; Figure 1). Across other subgroups, mDFS was numerically longer among patients who underwent resection alone (vs. resection + adjuvant therapy), completed adjuvant therapy (vs. incomplete adjuvant therapy), had *EGFR*m negative status (vs. positive), or an uncommon *EGFR*m (vs. common) (Table 3). At two years, the probability of remaining alive and disease free was 61% (95% CI: 55–67%) for all PD-L1+ patients and declined with advancing disease stage (stage IB: 84%, 95% CI: 77–92%; stage II: 53%, 95% CI: 44–64%; stage IIIA: 38%, 95% CI: 28–52%; see Supplementary Materials, Table S8). At four years, the probability of DFS was 44% (95% CI: 36–54%) overall, 68% (95% CI: 55–83%) for stage IB, 40% (95% CI: 29–56%) for stage II, and 17% (95% CI: 6–44%) for stage IIIA. Probabilities of DFS at four years were generally similar regardless of the level of PD-L1 expression (1–49% vs. ≥50%) and *EGFR*m status (Figure 2 and see Supplementary Materials, Table S8). As observed for OS, patients diagnosed with stage II or IIIA disease had a higher risk of recurrence and/or death within four years than those diagnosed with stage IB disease (stage II: HR 2.76, 95% CI: 1.69–4.51; stage IIIA: HR 4.29, 95% CI: 2.60–7.07).

## 4. Discussion

Contemporary data describing the characteristics, treatment, and outcomes of patients with resected, early-stage, PD-L1+ NSCLC are of interest given recently approved therapies, imminent approvals, and upcoming readouts from ongoing phase III clinical trials. However, published studies reporting on this population are currently limited. To our knowledge, this retrospective study is the first collaborative Canadian analysis to assess the characteristics and outcomes of these patients. The use of a real-world dataset derived from PALEOS enabled the conduct of specific analyses in the absence of limitations encountered with clinical trials, such as selection bias related to patient eligibility criteria.

The study results show that among the 539 patients who underwent PD-L1 testing, approximately 60% were positive for expression ≥ 1%, with about one-quarter having expression ≥ 50%. These proportions are similar to those reported in previous studies of various NSCLC populations, including those with advanced disease [28,29,30,31]. As expected, numerically higher proportions of PD-L1+ patients were smokers and had squamous cell histology compared with PD-L1–negative patients.

Among the PD-L1+ patients identified in this study, more than half had undergone resection alone without use of adjuvant therapy. This finding was somewhat unexpected given current treatment guideline recommendations [6,7,8,9], which support use of adjuvant therapy given the observed survival benefit in early-stage NSCLC [7,11,12]. However, the results may have been driven by the relatively high proportions of patients with stage IB disease (35%) and those aged ≥ 65 years (76%), who may have been too frail to receive adjuvant therapy. Previous Canadian studies have shown relatively low uptake of adjuvant therapy, particularly among elderly patients [32,33,34]. In addition, less than 5% of study patients had a macroscopic or microscopic residual tumor (R1/2) after surgical resection in both groups. This rate is lower than those reported in neoadjuvant and perioperative clinical trials of ICIs (R1/2 ~5–22%), which may reflect patient selection, patient preferences, and/or various approaches to such treatment [23,24,35]. Overall, the limited use of adjuvant therapy in this study could indicate a need for more effective perioperative systemic therapy strategies that offer an improved risk-benefit ratio for this real-world NSCLC population.

Reception of adjuvant therapy in the PD-L1+ cohort generally increased with advancing disease stage, though was still only received by 54% and 67% of PD-L1+ patients with stage II and IIIA disease, respectively. These results suggest that between 2016 and 2019, patient profiles and biomarker status did not have a strong impact on the decision to initiate adjuvant therapy at the three Canadian cancer centers. Another finding was that approximately one-third of the PD-L1+ patients experienced disease recurrence, a proportion consistent with that reported in other studies [36,37]; the majority of these events were distant metastases. Furthermore, among the 131 patients who received adjuvant therapy, 11 still had a locoregional and 50 had a metastatic first recurrence. This finding was not unexpected, given that patients receiving adjuvant therapy typically presented with more advanced disease; however, these results also suggest a need for more effective options.

In the PD-L1+ cohort, median follow-up was 22.6 months overall and longest for patients with stage IB disease at diagnosis (24.4 months). Considering this limited duration, it was not surprising that mOS was not reached in the overall cohort and most patient subgroups. As expected, the probability of survival at four years was higher among patients with stage IB disease than those in other stages; however, findings were similar regardless of PD-L1 expression (1–49% vs. ≥50%) and *EGFR*m status (positive vs. negative). Median DFS was 40 months in the overall PD-L1+ cohort but remained immature for most subgroups. With advancing stage, a clear trend was observed for declining mDFS, which was as low as 18.7 months among patients with stage IIIA disease. Similar to the results for OS, the four-year probabilities of DFS were similar irrespective of PD-L1 expression level and *EGFR*m positivity. Probabilities of both OS and DFS were generally similar at two years regardless of reception of adjuvant therapy, though were numerically higher at four years for those undergoing resection alone. These findings are again likely driven by the high proportion of stage IB patients who only received resection and the better prognosis of this population; furthermore, the sample size was much reduced at four years and 95% CIs were wide.

This real-world study of the characteristics and outcomes of patients with resected, early-stage PD-L1+ NSCLC provides useful insights that support use of new treatment options emerging for this population. Favorable clinical trial results have recently led to approval of adjuvant ICI therapies that were already well established in advanced NSCLC settings, with varying requirements for PD-L1 expression. For example, the phase III IMpower010 trial of adjuvant atezolizumab versus best supportive care showed an HR of 0.81 (95% CI: 0.67–0.99; *p* = 0.040) for DFS in the stage IB-IIIA, intention-to-treat (ITT) population [21]. Among stage II-IIIA patients with PD-L1 expression ≥1% and ≥50%, HRs were 0.66 (95% CI: 0.50–0.88; *p* = 0.0039) and 0.43 (95% CI: 0.27–0.68), respectively. On the basis of these results, atezolizumab was the first ICI approved for adjuvant use in resected, early-stage NSCLC, though indications in Canada and Europe are limited to patients with PD-L1 expression ≥ 50% and differ from that in the United States (US; expression ≥ 1%) [38,39,40]. Results from the phase III PEARLS/KEYNOTE-091 trial have also been reported, showing significant improvement of DFS with adjuvant pembrolizumab compared with placebo in an all-comers population for PD-L1 expression with resected stage IB-IIIA NSCLC (HR 0.81, 95% CI: 0.68–0.96) [22]; the benefit was only observed among those who also received adjuvant chemotherapy. Adjuvant pembrolizumab is approved for use after resection and chemotherapy in Canada, the US, and Europe, without restriction related to PD-L1 expression [41,42,43]. Additionally, nivolumab used in combination with chemotherapy has been approved for neoadjuvant use in NSCLC based on the phase III CheckMate-816 trial, which reported an HR for disease progression, recurrence, or death of 0.63 (97.38% CI: 0.43–0.91, *p* = 0.005) [23]. The therapy’s indications have no restriction related to PD-L1 expression level in Canada and the US [44,45], while expression ≥ 1% is required in Europe [46].

Even more recently, phase III trials have also shown the benefit of ICIs in the perioperative setting of NSCLC. At 36.6 months of follow-up in KEYNOTE-671, median event-free survival (mEFS) was significantly improved with pembrolizumab + chemotherapy compared with placebo + chemotherapy (47.2 vs. 18.3 months; HR, 0.59 (95% CI: 0.48–0.72)) [24,25]. This trial also showed a significant OS advantage with the pembrolizumab combination (HR, 0.72 (95% CI: 0.56–0.93); *p* = 0.00517), which subsequently became the first perioperative regimen approved for early-stage NSCLC [41,43]. Similarly, at a median follow-up of 11.7 months in the AEGEAN trial, mEFS was not reached in the durvalumab + chemotherapy arm but was 25.9 months in the chemotherapy alone arm, a significant 32% reduction in the risk of disease progression precluding definitive surgery, disease recurrence, or death (stratified HR, 0.68 (95% CI: 0.53–0.88); *p* = 0.004) [26]. Data from the perioperative CheckMate 77T trial of nivolumab + chemotherapy have also shown significant improvement of EFS versus chemotherapy + placebo at a median follow-up of 25.4 months (not reached vs. 18.4 months; HR, 0.58 (97.36% CI: 0.42–0.81); *p* < 0.001) [27]. The Neotorch trial, which was primarily conducted in China, has shown similarly encouraging results [35]. In relation to the current study, it is relevant to note that patients with known *EGFR*m were either excluded from these neoadjuvant, adjuvant, and perioperative trials, or outcomes were evaluated among very few *EGFR*m-positive patients, limiting insights on this population. Numerous other phase III ICI trials remain ongoing in resectable, early-stage NSCLC, such as the ALCHEMIST CHEMO IO (ACCIO; A081801) [47] trial of pembrolizumab and the NADIM-ADJUVANT [48] and ALCHEMIST-nivo (ANVIL; EA5142) [49] trials of nivolumab. The current study’s findings underscore the needs experienced by patients who may be eligible for these treatment options.

This study has both strengths and limitations. A key strength was the evaluation of a relatively large dataset that reflects real-world patient information, thereby permitting analysis without the restrictions of the clinical trial setting. Moreover, data were derived from three large community and academic cancer centers with variability in patient characteristics, ethnicity, and socioeconomic status, which may allow generalizability to other North American populations. Still, the use of data from these highly specialized cancer centers, which were located within one province and country, may have introduced bias related to patient selection and local treatment practices. Furthermore, although every effort was taken to ensure that information was correctly abstracted from clinical notes, some patient records were incomplete. Data for disease staging were available at the time of patient diagnosis but not at the initiation of treatment; accordingly, some patients may have had more advanced disease than the rates reported herein. Any treatment provided outside of oncology clinics was not captured, potentially leading to misclassification of treatment patterns and outcomes. Additionally, given the timing of study conduct, our analysis focused on PD-L1+ patients; however, as recent ICI trials (and drug approvals) indicate a treatment benefit among patients with PD-L1–negative disease, this population could be explored in future analyses. DFS was included as an exploratory objective as it required interpretation of clinical notes to identify disease recurrence; the proportion of recurrences identified on the basis of routine scans versus symptom onset is unknown but may have influenced this outcome. Finally, given that some patients may achieve cure, the average follow-up was relatively short for this resected, early-stage NSCLC population, and therefore future studies should aim to address this gap.

## 5. Conclusions

This real-world study of patients with resected, early-stage NSCLC found that the prevalence of PD-L1 expression (≥1%) was similar to that reported in previous studies, including those conducted in the metastatic setting. The use of adjuvant chemotherapy was lower than expected. Over the study’s four-year timeframe, approximately one-third of patients experienced disease recurrence despite resection ± adjuvant therapy, which frequently occurred in the lung and CNS. As expected, patients diagnosed with stage IB disease had a higher probability of survival than those with stage II or IIIA disease; DFS varied across subgroups but was generally poor. At four years, survival outcomes were similar regardless of the level of PD-L1 expression or *EGFR*m status. These findings indicate that although cure is possible for some patients after resection ± adjuvant therapy, recurrence remains common, necessitating treatment in the metastatic setting and leading to poor survival. This study provides important context regarding the experience of the resected, early-stage PD-L1+ NSCLC population, and emphasizes the need for access to neoadjuvant, adjuvant, and perioperative regimens that can potentially improve patient outcomes.

## Figures and Tables

**Table 1:** Demographic and clinical characteristics, PD-L1 prevalence cohort (N = 539).

Characteristic	PD-L1 Negative	PD-L1 Positive ^1^	All PD-L1–Tested Patients
Overall, n (% of 539)	222 (41.2)	317 (58.8)	539 (100.0)
PD-L1 Expression, n (%)			
1–49%	--	180 (56.8)	180 (33.4)
≥50%	--	137 (43.2)	137 (25.4)
Stage at Diagnosis, n (%)			
IB	98 (44.1)	112 (35.3)	210 (39.0)
II	83 (37.4)	126 (39.8)	209 (38.8)
IIIA	41 (18.5)	79 (24.9)	120 (22.3)
Sex, n (%)			
Female	116 (52.2)	162 (51.1)	278 (51.6)
Male	106 (47.8)	155 (48.9)	261 (48.4)
Race ^2^, n (%)			
Asian, NOS	18 (8.1)	13 (4.1)	31 (5.8)
Caucasian	20 (9.0)	44 (13.9)	64 (11.9)
East, Central, or South Asian	17 (7.7)	19 (6.0)	36 (6.7)
Other	45 (20.3)	81 (25.5)	126 (23.4)
Unknown	122 (55.0)	160 (50.5)	282 (52.3)
Smoking Status, n (%)			
Current/former smoker	145 (65.3)	270 (85.2)	415 (77.0)
Never smoker	66 (29.7)	42 (13.2)	108 (20.0)
Unknown	11 (5.0)	5 (1.6)	16 (3.0)
Histopathological Diagnosis, n (%)			
Adenocarcinoma	173 (77.9)	198 (62.4)	371 (68.8)
Squamous cell carcinoma	30 (13.5)	87 (27.4)	117 (21.7)
Adenosquamous carcinoma	0 (0.0)	9 (2.8)	9 (1.7)
Large cell carcinoma	12 (5.4)	2 (0.6)	14 (2.6)
Other ^3^	7 (3.2)	21 (6.6)	28 (5.2)
*EGFR*m Testing Status, n (%)			
Unknown/Not tested	53 (23.9)	96 (30.3)	149 (27.6)
Known/Tested ^4^	169 (76.1)	221 (69.7)	390 (72.4)
*EGFR*m Status ^5^, n (%)			
Negative	123 (72.8)	185 (83.7)	308 (79.0)
Positive	46 (27.2)	36 (16.3)	82 (21.0)
Common Exon 19 deletion	20 (11.8)	14 (6.3)	34 (8.7)
Common Exon 21 *L858R*	17 (10.1)	9 (4.1)	26 (6.7)
Uncommon mutations	9 (5.3)	13 (5.9)	22 (5.6)

^1^ PD-L1 expressed at ≥1%. ^2^ Categorized based on patient self-identification and physician reporting; data not available from LHSC. ^3^ Among all tested patients, other histopathological diagnoses included mixed morphology (n = 6), NSCLC NOS (n = 5), pleomorphic carcinoma (n = 2), desmoplastic (n = 1), and other NOS (n = 14). Among PD-L1+ patients, other histopathological diagnoses included mixed morphology (n = 5), NSCLC NOS (n = 4), pleomorphic carcinoma (n = 2), desmoplastic (n = 1), and other NOS (n = 9). ^4^ *EGFR*m testing results were available for 390 patients: 376/422 non-squamous and 14/117 squamous cell carcinoma patients. ^5^ Calculations based on the following denominators for *EGFR*m-tested patients with results: 169 PD-L1–negative, 221 PD-L1+, and 390 total patients. CI, confidence interval; *EGFR*m, epidermal growth factor receptor mutation; LHSC, London Health Sciences Centre; NOS, not otherwise specified; NR, not reported; NSCLC, non-small cell lung cancer; PD-L1, programmed cell death-ligand 1.

**Table 2:** Demographic and clinical characteristics stratified by initial treatment type, PD-L1+ cohort (N = 317).

Characteristic	Resection Alone	Resection + Adjuvant Therapy	All PD-L1+ Patients
Overall, n (% of 317)	186 (58.7)	131 (41.3)	317 (100)
Age at Diagnosis			
Years, mean (SD)	71.8 (8.7)	68.0 (8.5)	70.2 (8.8)
≥65 years, n (%)	149 (80.1)	92 (70.2)	241 (76.0)
<65 years, n (%)	37 (19.9)	39 (29.8)	76 (24.0)
Stage at Diagnosis, n (%)			
IB	102 (54.8)	10 (7.6)	112 (35.3)
II	58 (31.0)	68 (51.9)	126 (39.8)
IIIA	26 (13.9)	53 (40.4)	79 (24.9)
Sex			
Female	97 (52.2)	65 (49.6)	162 (51.1)
Male	89 (47.8)	66 (50.4)	155 (48.9)
PD-L1 Expression, n (%)			
1–49%	105 (56.5)	75 (57.3)	180 (56.8)
≥50%	81 (43.5)	56 (42.7)	137 (43.2)
Race ^1^, n (%)			
Asian, NOS	6 (3.2)	7 (5.3)	13 (4.1)
Caucasian	20 (10.8)	24 (18.3)	44 (13.9)
East, Central, or South Asian	9 (4.8)	10 (7.6)	19 (6.0)
Other	54 (29.0)	27 (20.6)	81 (25.6)
Unknown	97 (52.2)	63 (48.1)	160 (50.5)
Weight Category, n (%)			
<60 kg	40 (21.5)	20 (15.3)	60 (18.9)
≥60 kg	111 (59.7)	92 (70.2)	203 (64.0)
Unknown	35 (18.8)	19 (14.5)	54 (17.0)
Smoking Status, n (%)			
Current/former smoker	164 (88.2)	106 (80.9)	270 (85.2)
Never smoker	18 (9.7)	24 (18.3)	42 (13.3)
Unknown	4 (2.2)	1 (0.1)	5 (1.6)
ECOG Status, n (%)			
0	69 (37.1)	32 (24.4)	101 (31.9)
1	31 (16.7)	29 (22.1)	60 (18.9)
2	1 (0.5)	1 (0.8)	2 (0.6)
Unknown	85 (45.7)	69 (52.7)	154 (48.6)
Result of Surgery, n (%)			
R0	180 (96.8)	124 (94.7)	304 (95.9)
R1	6 (3.2)	5 (3.9)	11 (3.5)
R2	0 (0.0)	2 (1.5)	2 (0.6)
Histopathological Diagnosis, n (%)			
Adenocarcinoma	110 (59.1)	88 (67.2)	198 (62.5)
Squamous cell carcinoma	53 (28.5)	34 (30.0)	87 (27.4)
Adenosquamous carcinoma	6 (3.2)	3 (2.3)	9 (2.8)
Large cell carcinoma	2 (1.1)	0 (0.0)	2 (0.6)
Other	15 (8.1)	6 (4.6)	21 (6.6)
*EGFR*m Testing Status, n (%)			
Unknown/Not tested	61 (32.8)	35 (26.7)	96 (30.3)
Known/Tested ^2^	125 (67.2)	96 (73.3)	221 (69.7)
*EGFR*m Status ^3^, n (%)			
Negative	110 (88.0)	75 (78.1)	185 (83.7)
Positive	15 (12.0)	21 (21.9)	36 (16.3)
Common Exon 19 deletion	6 (4.8)	8 (8.3)	14 (6.3)
Common Exon 21 *L858R*	3 (2.4)	6 (6.3)	9 (4.1)
Uncommon mutations	6 (4.8)	7 (7.3)	13 (5.9)

^1^ Categorized based on self-identification and physician reporting; data not available from LHSC. ^2^ *EGFR*m testing results were available for 221 patients: 212/230 non-squamous and 9/87 squamous cell carcinoma patients. ^3^ Calculations based on the following denominators for *EGFR*m-tested patients with results: 125 with resection alone, 96 with resection + adjuvant therapy, 221 total patients. ECOG, Eastern Cooperative Oncology Group; *EGFR*m, epidermal growth factor receptor mutation; LHSC, London Health Sciences Centre; NOS, not otherwise specified; PD-L1, programmed cell death-ligand 1; SD, standard deviation.

**Table 3:** Median OS and median DFS, PD-L1+ cohort (N = 317).

Variable	No. of Patients	No. of Deaths	Median OS mo. (95% CI)	No. of DFS Events	Median DFS mo. (95% CI)
Overall	317	66	NE (NE–NE)	125	40.0 (30.0–NE)
Stage at Diagnosis					
IB	112	11	NE (NE–NE)	23	NE (NE–NE)
II	126	31	45.5 (40.9–NE)	53	30.0 (19.6–NE)
IIIA	79	24	NE (33.3–NE)	49	18.7 (12.7–29.5)
II and IIIA combined	205	55	NE (40.9–NE)	102	22.6 (19.1–32.9)
Result of Surgery					
R0	304	62	NE (NE–NE)	118	40.9 (30.0–NE)
R1	11	3	37.2 (37.2–NE)	6	19.4 (16.9–NE)
R2	2	1	22.6 (NE–NE)	1	12.0 (12.0–NE)
R1 + R2	13	4	37.2 (22.6–NE)	7	19.4 (12.7–NE)
Treatment Type					
Resection alone	186	33	NE (NE–NE)	58	NE (40.0–NE)
Resection + adjuvant therapy (systemic and/or RT)	131	33	NE (45.5–NE)	67	28.2 (20.6–42.2)
Adjuvant Therapy Status (systemic and/or RT)					
Complete	86	20	NE (45.5–NE)	43	32.0 (22.6–NE)
Incomplete ^1^	38	10	NE (33.1–NE)	21	21.4 (10.1–NE)
PD-L1 Expression					
1–49%	180	33	NE (NE–NE)	70	38.9 (30.0–NE)
≥50%	137	33	NE (NE–NE)	55	40.0 (22.5–NE)
*EGFR*m Status					
Positive	36	9	NE (38.9–NE)	18	32.9 (18.7–NE)
Common mutations	23	5	NE (33.3–NE)	11	22.5 (14.1–NE)
Uncommon mutations	13	3	NE (38.9–NE)	6	38.9 (16.9–NE)
Negative	185	31	NE (NE–NE)	67	42.2 (30.5–NE)
Unknown	96	26	NE (NE–NE)	40	40.0 (19.6–NE)

Note: Some outcomes were NE due to an insufficient number of events. ^1^ Adjuvant therapy was not completed; explanation was not recorded. A total of 7/131 patients receiving adjuvant therapy could not be classified as complete or incomplete. CI, confidence interval; *EGFR*m, epidermal growth factor receptor mutation; DFS, disease-free survival; mo., months; NE, not estimable; No., number; OS, overall survival; PD-L1, programmed cell death-ligand 1; RT, radiation therapy.

## Data Availability

The data presented in this study are available on request from the corresponding author due to the storage of data in a registry for which Pulse Infoframe is the custodian. Please note that the corresponding author needs to make a request to Pulse Infoframe to obtain the dataset.

## Supplementary Materials

The following supporting information can be downloaded at: https://www.mdpi.com/article/10.3390/curroncol31110497/s1: Figure S1: Overall survival (A) and DFS (B) among PD-L1+ patients stratified by *EGFR*m status; Table S1: Demographic and clinical characteristics stratified by initial treatment type among PD-L1+/*EGFR*m-negative (wild type) patients; Table S2: Frequency of first disease recurrence, PD-L1+ cohort; Table S3: Sites of first disease recurrence, PD-L1+ cohort; Table S4: Type of adjuvant treatment, PD-L1+ cohort; Table S5: Type of adjuvant treatment received among patients with known *EGFR*m status, PD-L1+ cohort; Table S6: Type of treatment after first metastatic recurrence, PD-L1+ cohort; Table S7: Overall survival at 2 and 4 years, PD-L1+ cohort; Table S8: Disease-free survival at 2 and 4 years, PD-L1+ cohort.

## References

[1] Bray F., Laversanne M., Sung H., Ferlay J., Siegel R.L., Soerjomataram I., Jemal A. (2024). Global cancer statistics 2022: GLOBOCAN estimates of incidence and mortality worldwide for 36 cancers in 185 countries. CA Cancer J. Clin..

[2] Canadian Cancer Society Lung and Bronchus Cancer Statistics. Last Updated May 2024. https://cancer.ca/en/cancer-information/cancer-types/lung/statistics.

[3] Canadian Cancer Statistics Advisory Committee (2020). Canadian Cancer Statistics: A 2020 Special Report on Lung Cancer. Canadian Cancer Society. https://cdn.cancer.ca/-/media/files/cancer-information/resources/publications/2020-canadian-cancer-statistics-special-report/2020-canadian-cancer-statistics-special-report-en.pdf.

[4] Cagle P.T., Allen T.C., Olsen R.J. (2013). Lung cancer biomarkers: Present status and future developments. Arch. Pathol. Lab. Med..

[5] Le Chevalier T. (2010). Adjuvant chemotherapy for resectable non-small-cell lung cancer: Where is it going?. Ann. Oncol..

[6] Postmus P.E., Kerr K.M., Oudkerk M., Senan S., Waller D.A., Vansteenkiste J., Escriu C., Peters S. (2017). Early and locally advanced non-small-cell lung cancer (NSCLC): ESMO Clinical Practice Guidelines for diagnosis, treatment and follow-up. Ann. Oncol..

[7] Kris M.G., Gaspar L.E., Chaft J.E., Kennedy E.B., Azzoli C.G., Ellis P.M., Lin S.H., Pass H.I., Seth R., Shepherd F.A. (2017). Adjuvant systemic therapy and adjuvant radiation therapy for stage I to IIIA completely resected non-small-cell lung cancers: American Society of Clinical Oncology/Cancer Care Ontario Clinical Practice Guideline update. J. Clin. Oncol..

[8] National Comprehensive Cancer Network (2024). NCCN Clinical Practice Guidelines in Oncology. Non-small Cell Lung Cancer.

[9] Provincial Health Services Authority BC Cancer. Management of Non-Small Cell Lung Cancer. Last Updated: February 2008. http://www.bccancer.bc.ca/health-professionals/clinical-resources/cancer-management-manual/lung/lung#Non-Small-Cell-Lung-Cancer-(NSCLC).

[10] Lim J.U., Yeo C.D. (2022). Update on adjuvant therapy in completely resected NSCLC patients. Thorac. Cancer.

[11] Pignon J.P., Tribodet H., Scagliotti G.V., Douillard J.Y., Shepherd F.A., Stephens R.J., Dunant A., Torri V., Rosell R., Seymour L. (2008). Lung adjuvant cisplatin evaluation: A pooled analysis by the LACE Collaborative Group. J. Clin. Oncol..

[12] Bradbury P., Sivajohanathan D., Chan A., Kulkarni S., Ung Y., Ellis P.M. (2017). Postoperative adjuvant systemic therapy in completely resected non-small-cell lung cancer: A systematic review. Clin. Lung Cancer.

[13] Taylor M.D., Nagji A.S., Bhamidipati C.M., Theodosakis N., Kozower B.D., Lau C.L., Jones D.R. (2012). Tumor recurrence after complete resection for non-small cell lung cancer. Ann. Thorac. Surg..

[14] Uramoto H., Tanaka F. (2014). Recurrence after surgery in patients with NSCLC. Transl. Lung Cancer Res..

[15] Felip E., Rosell R., Maestre J.A., Rodríguez-Paniagua J.M., Morán T., Astudillo J., Alonso G., Borro J.M., González-Larriba J.L., Torres A. (2010). Preoperative chemotherapy plus surgery versus surgery plus adjuvant chemotherapy versus surgery alone in early-stage non-small-cell lung cancer. J. Clin. Oncol..

[16] Deslypere G., Gullentops D., Wauters E., Vansteenkiste J. (2018). Immunotherapy in non-metastatic non-small cell lung cancer: Can the benefits of stage IV therapy be translated into earlier stages?. Ther. Adv. Med. Oncol..

[17] Broderick S.R. (2020). Adjuvant and neoadjuvant immunotherapy in non-small cell lung cancer. Thorac. Surg. Clin..

[18] Wu Y.L., Tsuboi M., He J., John T., Grohe C., Majem M., Goldman J.W., Laktionov K., Kim S.W., Kato T. (2020). Osimertinib in resected EGFR-mutated non-small-cell lung cancer. N. Engl. J. Med..

[19] Remon J., Soria J.-C., Peters S., On behalf of the ESMO Guidelines Committee (2021). Early and locally advanced non-small-cell lung cancer: An update of the ESMO Clinical Practice Guidelines focusing on diagnosis, staging and systemic and local therapy. Ann. Oncol..

[20] Pisters K., Kris M.G., Gaspar L.E., Ismaila N. (2022). Adjuvant systemic therapy and adjuvant radiation therapy for stage I-IIIA completely resected non–small-cell lung cancer: ASCO guideline rapid recommendation update. J. Clin. Oncol..

[21] Felip E., Altorki N., Zhou C., Csoszi T., Vynnychenko I., Goloborodko O., Luft A., Akopov A., Martinez-Marti A., Kenmotsu H. (2021). Adjuvant atezolizumab after adjuvant chemotherapy in resected stage IB-IIIA non-small-cell lung cancer (IMpower010): A randomised, multicentre, open-label, phase 3 trial. Lancet.

[22] Besse B., Havel L., Peters S., Marreaud S.I., Jha N., Oselin K., Gonzalez E.E., Casado M.D.I., Martinez-Marti A., Faehling M. (2023). 120MO—Adjuvant pembrolizumab versus placebo for early-stage NSCLC after resection and optional chemotherapy: Updated results From PEARLS/KEYNOTE-091. Ann. Oncol..

[23] Forde P.M., Spicer J., Lu S., Provencio M., Mitsudomi T., Awad M.M., Felip E., Broderick S.R., Brahmer J.R., Swanson S.J. (2022). Neoadjuvant nivolumab plus chemotherapy in resectable lung cancer. N. Engl. J. Med..

[24] Wakelee H., Liberman M., Kato T., Tsuboi M., Lee S.H., Gao S., Chen K.N., Dooms C., Majem M., Eigendorff E. (2023). Perioperative pembrolizumab for early-stage non-small-cell lung cancer. N. Engl. J. Med..

[25] Spicer J.D., Gao S., Liberman M., Kato T., Tsuboi M., Lee S.H., Chen K.N., Dooms C., Majem M., Eigendorff E. (2023). LBA56 Overall survival in the KEYNOTE-671 study of perioperative pembrolizumab for early-stage non-small-cell lung cancer (NSCLC). Ann. Oncol..

[26] Heymach J.V., Harpole D., Mitsudomi T., Taube J.M., Galffy G., Hochmair M., Winder T., Zukov R., Garbaos G., Gao S. (2023). Perioperative durvalumab for resectable non–small-cell lung cancer. N. Eng. J. Med..

[27] Cascone T., Awad Mark M., Spicer Jonathan D., He J., Lu S., Sepesi B., Tanaka F., Taube Janis M., Cornelissen R., Havel L. (2024). Perioperative nivolumab in resectable lung cancer. N. Eng. J. Med..

[28] Aggarwal C., Abreu D.R., Felip E., Carcereny E., Gottfried M., Wehler T., Ahn M.J., Dolled-Filhart M., Zhang J., Shentu Y. (2016). Prevalence of PD-L1 expression in patients with non-small cell lung cancer screened for enrollment in KEYNOTE-001, -010, and -024. Ann. Oncol..

[29] Cruz-Rico G., Aviles-Salas A., Popa-Navarro X., Lara-Mejia L., Catalan R., Sanchez-Reyes R., Lopez-Sanchez D., Cabrera-Miranda L., Aquiles Maldonado-Martinez H., Samtani-Bassarmal S. (2021). Association of lung adenocarcinoma subtypes according to the IASLC/ATS/ERS classification and programmed cell death ligand 1 (PD-L1) expression in tumor cells. Pathol. Oncol. Res..

[30] Dietel M., Savelov N., Salanova R., Micke P., Bigras G., Hida T., Antunez J., Guldhammer Skov B., Hutarew G., Sua L.F. (2019). Real-world prevalence of programmed death ligand 1 expression in locally advanced or metastatic non-small-cell lung cancer: The global, multicenter EXPRESS study. Lung Cancer.

[31] Holmes M., Mahar A., Lum T., Boyer M., Kao S., Cooper W. (2019). P1.09-26 Prevalence of PD-L1 expression rates in different NSCLC specimens. J. Thorac. Oncol..

[32] Cuffe S., Booth C.M., Peng Y., Darling G.E., Li G., Kong W., Mackillop W.J., Shepherd F.A. (2012). Adjuvant chemotherapy for non-small-cell lung cancer in the elderly: A population-based study in Ontario, Canada. J. Clin. Oncol..

[33] Booth C.M., Shepherd F.A., Peng Y., Darling G., Li G., Kong W., Mackillop W.J. (2012). Adjuvant chemotherapy for non-small cell lung cancer: Practice patterns and outcomes in the general population of Ontario, Canada. J. Thorac. Oncol..

[34] Evans W.K., Stiff J., Woltman K.J., Ung Y.C., Su-Myat S., Manivong P., Tsang K., Nazen-Rad N., Gatto A., Tyrrell A. (2017). How equitable is access to treatment for lung cancer patients? A population-based review of treatment practices in Ontario. Lung Cancer Manag..

[35] Lu S., Zhang W., Wu L., Wang W., Zhang P., Fang W., Xing W., Chen Q., Yang L., Mei J. (2024). Perioperative toripalimab plus chemotherapy for patients with resectable non-small cell lung cancer: The Neotorch randomized clinical Trial. JAMA.

[36] Nakagawa M., Uramoto H., Oka S., Chikaishi Y., Iwanami T., Shimokawa H., So T., Hanagiri T., Tanaka F. (2012). Clinical significance of IGF1R expression in non-small-cell lung cancer. Clin. Lung Cancer.

[37] Yamashita T., Uramoto H., Onitsuka T., Ono K., Baba T., So T., So T., Takenoyama M., Hanagiri T., Oyama T. (2010). Association between lymphangiogenesis-/micrometastasis- and adhesion-related molecules in resected stage I NSCLC. Lung Cancer.

[38] Hoffman-La Roche Product Monograph: TECENTRIQ (Atezolizumab for Injection). Revised: 15 March 2024. https://assets.roche.com/f/173850/x/4558b62072/tecentriq_pm_cie.pdf.

[39] European Medicines Agency Summary of Product Characteristics: TECENTRIQ. Last Updated: 25 March 2024. https://www.ema.europa.eu/en/medicines/human/EPAR/tecentriq.

[40] Food and Drug Administration Highlights of Prescribing Information for TECENTRIQ (Atezolizumab). Revised: April 2024. https://www.accessdata.fda.gov/drugsatfda_docs/label/2024/761034s053lbl.pdf.

[41] Food and Drug Administration Highlights of Prescribing Information for KEYTRUDA (Pembrolizumab). Revised: March 2024. https://www.accessdata.fda.gov/drugsatfda_docs/label/2024/125514s160lbl.pdf.

[42] Merck Product Monograph: KEYTRUDA (Pembrolizumab). Date of Revision: 12 April 2024. https://www.merck.ca/en/wp-content/uploads/sites/20/2021/04/KEYTRUDA-PM_E.pdf.

[43] European Medicines Agency Summary of Product Characteristics: KEYTRUDA. Last Updated: 13 May 2024. https://www.ema.europa.eu/en/documents/product-information/keytruda-epar-product-information_en.pdf.

[44] Bristol-Myers Squib Product Monograph: OPDIVO (Nivolumab) for Injection. Revised: 29 December 2023. https://www.bms.com/assets/bms/ca/documents/productmonograph/OPDIVO_EN_PM.pdf.

[45] Food and Drug Administration Highlights of Prescribing Information for OPDIVO (Nivolumab). Revised: March 2024. https://www.accessdata.fda.gov/drugsatfda_docs/label/2022/125554s112lbl.pdf.

[46] European Medicines Agency Summary of Product Characteristics: OPDIVO. Last Updated: 4 April 2024. https://www.ema.europa.eu/en/documents/product-information/opdivo-epar-product-information_en.pdf.

[47] Sands J.M., Mandrekar S.J., Kozono D., Oxnard G.R., Hillman S.L., Wigle D.A., Govindan R., Carlisle J., Gray J., Salama J.K. (2021). Integration of immunotherapy into adjuvant therapy for resected non-small-cell lung cancer: ALCHEMIST chemo-IO (ACCIO). Immunotherapy.

[48] Calvo V., Domine M., Sullivan I., Gonzalez-Larriba J.-L., Ortega A.L., Bernabé R., Sala M.A., Campos B., Castro J.D., Martín-Martorell P. (2021). A phase III clinical trial of adjuvant chemotherapy versus chemoimmunotherapy for stage IB-IIIA completely resected non-small cell lung cancer (NSCLC) patients nadim-adjuvant: New adjuvant trial of chemotherapy versus chemoimmunotherapy. J. Clin. Oncol..

[49] Chaft J.E., Dahlberg S.E., Khullar O.V., Edelman M.J., Simone C.B., Heymach J., Rudin C.M., Ramalingam S.S. (2018). EA5142 adjuvant nivolumab in resected lung cancers (ANVIL). J. Clin. Oncol..

