# Supplementary material for: Programmed Cell Death-Ligand 1 Expression and Clinical Outcomes Among Patients with Resected, Early-Stage Non-Small Cell Lung Cancer: A Real-World Study
Source: Curr Oncol. 2024 Oct 31;31(11):6735–48. doi: 10.3390/curroncol31110497 (PMC11593080; doi:10.3390/curroncol31110497)
Supplement: Supplementary file 1 [file curroncol-31-00497-s001.zip › curroncol-3200358-supplementary.pdf]

Programmed cell death-ligand 1 expression and clinical outcomes among patients with resected, early-stage non-small cell lung cancer: a real-world study

Parneet K. Cheema, Iqra Syed, Femida Gwadry-Sridhar, Muhammad Rakibuz-Zaman, Robin Sachdeva, Alec Pencz, Luna Zhan, Katrina Hueniken, Devalben Patel, Karmugi Balaratnam, Khaleeq Khan, Benjamin Grant, Brandon S. Sheffield, M. Elizabeth O. Locke, Daniel Moldaver, Mary Kate Shanahan, Geoffrey Liu, M. Sara Kuruvilla

Table S1. Demographic and clinical characteristics stratified by initial treatment type among PD-L1+/*EGFR*m-negative (wild type) patients

**Characteristic**	**Resection Alone**	**Resection + Adjuvant Therapy**	**All PD-L1+/*EGFR*m wt Patients**
Overall, n (% of 185)	110 (59.5)	75 (40.5)	185 (100.0)
Mean Age at Diagnosis			
Years (SD)	71.5 (8.1)	67.9 (9.2)	70.0 (8.7)
Stage at Diagnosis, n (%)			
IB	66 (60.0)	7 (9.3)	73 (39.5)
II	31 (28.2)	39 (52.0)	70 (37.8)
IIIA	13 (11.8)	29 (38.7)	42 (22.7)
Sex, n (%)			
Female	63 (57.3)	45 (60.0)	108 (58.4)
Male	47 (42.7)	30 (40.0)	77 (41.6)
Race[1], n (%)			
Asian, NOS	2 (1.8)	4 (5.3)	6 (3.2)
Caucasian	11 (10.0)	12 (16.0)	23 (12.4)
East, Central, or South Asian	4 (3.6)	3 (4.0)	7 (3.8)
Other	35 (31.8)	18 (24.0)	53 (28.6)
Unknown	58 (52.7)	38 (50.7)	96 (51.9)
Weight Category, n (%)			
<60 kg	27 (24.5)	14 (18.7)	41 (22.2)
≥60 kg	62 (56.4)	49 (65.3)	111 (60.0)
Unknown	21 (19.1)	12 (16.0)	33 (17.8)
Smoking Status, n (%)			
Current/former smoker	99 (90.0)	63 (84.0)	162 (87.6)
Never smoker	8 (7.3)	12 (16.0)	20 (10.8)
Unknown	3 (2.7)	0 (0.0)	3 (1.6)
ECOG Status, n (%)			
0	45 (40.9)	20 (26.7)	65 (35.1)
1	17 (15.5)	19 (25.3)	36 (19.5)
2	1 (0.9)	1 (1.3)	2 (1.1)
Unknown	47 (42.7)	35 (46.7)	82 (44.3)
Result of Surgery, n (%)			
R0	107 (97.3)	71 (94.7)	178 (96.2)
R1	3 (2.7)	4 (5.3)	7 (3.8)
Recurrence Type, n (%)			
Locoregional	11 (10.0)	5 (6.7)	16 (8.6)
Metastatic	16 (14.5)	27 (36.0)	43 (23.2)
No recurrence	83 (75.5)	43 (57.3)	126 (68.1)

[1] Categorized based on patient self-identification and physician reporting.

ECOG, Eastern Cooperative Oncology Group; *EGFR*m, epidermal growth factor receptor mutation; NOS, not otherwise specified; NR, not reported; PD-L1, programmed cell death-ligand 1; SD, standard deviation; wt, wild type.

Table S2. Frequency of first disease recurrence, PD-L1+ cohort

Characteristic	Resection Alone n = 186	Resection + Adjuvant Therapy n = 131	Total N = 317
Recurrence, n (%)	40 (21.5)	61 (46.6)	101 (31.9)
Locoregional (n [%] of those with recurrence)	19 (47.5)	11 (18.0)	30 (29.7)
Metastatic (n [%] of those with recurrence)	21 (52.5)	50 (82.0)	71 (70.3)
No recurrence, n (%)	146 (78.5)	70 (53.4)	216 (68.1)

PD-L1, programmed cell death-ligand 1.

Table S3. Sites of first disease recurrence, PD-L1+ cohort

Site of Recurrence	Frequency, n (%)
CNS	19 (14.7)
Lung	25 (19.4)
Lymph nodes (distant or locoregional)	36 (27.9)
Pleural/Pericardial	11 (8.5)
Bone	8 (6.2)
Liver	8 (6.2)
Adrenal	4 (3.1)
Chest wall	3 (2.3)
Other – not specified	15 (11.6)
Total number of sites of first recurrence	129

Note: data are not mutually exclusive (i.e., patients may have experienced first recurrence at >1 site).
CNS, central nervous system; PD-L1, programmed cell death-ligand 1.

Table S4. Type of adjuvant treatment, PD-L1+ cohort

**Adjuvant Treatment Type**<br>**n (%)**	**Total**<br>**N = 131**
Chemotherapy	103 (78.6)
Chemotherapy + ICI[a]	2 (1.5)
Chemotherapy + RT	5 (3.8)
Chemotherapy + ICI[a] + RT	2 (1.5)
ICI[1] + RT	1 (0.8)
RT	18 (13.7)

[1] ICI therapy received within a clinical trial.
ICI, immune checkpoint inhibitor; PD-L1, programmed cell death-ligand 1; RT, radiation therapy.

Table S5. Type of adjuvant treatment received among patients with known *EGFR*m status, PD-L1+ cohort

**Adjuvant Treatment Type n (column %)**	***EGFR*m Negative n = 75**	***EGFR*m Positive n = 21**	**Total N = 96**
Chemotherapy	58 (77.3)	15 (71.4)	73 (76.0)
Chemotherapy + RT	5 (6.7)	0 (0.0)	5 (5.2)
Chemotherapy + ICI[a] + RT	1 (1.3)	1 (4.8)	2 (2.1)
ICI[1] + RT	1 (1.3)	0 (0.0)	1 (1.0)
RT	10 (13.3)	5 (23.8)	15 (15.6)

[1] ICI therapy received within a clinical trial.

*EGFR*m, epidermal growth factor receptor mutation; ICI, immune checkpoint inhibitor; PD-L1, programmed cell death-ligand 1; RT, radiation therapy.

Table S6. Type of treatment after first metastatic recurrence, PD-L1+ cohort

**Treatment Type**[1] **n (%)**	**Total N = 71**	**Patients with Known Treatment Type n = 57**
**Chemotherapy ± RT**	**4 (5.6)**	**4 (7.0)**
Chemotherapy	2 (2.8)	2 (3.5)
Chemotherapy + RT	2 (2.8)	2 (3.5)
**Chemotherapy + ICI ± RT**	**3 (4.2)**	**3 (5.3)**
Chemotherapy + ICI	2 (2.8)	2 (3.5)
Chemotherapy + ICI + RT	1 (1.4)	1 (1.8)
**ICI ± RT**	**14 (19.7)**	**14 (24.6)**
ICI	9 (12.6)	9 (15.8)
ICI + RT	5 (7.0)	5 (8.8)
RT	16 (22.5)	16 (28.1)
Surgery	1 (1.4)	1 (1.8)
Surgery + RT	3 (4.2)	3 (5.3)
EGFR-TKI	7 (9.9)	7 (12.3)
No treatment	8 (11.3)	8 (14.0)
Unknown	14 (19.7)	--
Other	1 (1.4)	1 (1.8)

[1] First treatment received after first metastatic recurrence.

ICI, immune checkpoint inhibitor; EGFR-TKI, epidermal growth factor receptor tyrosine kinase inhibitor; PD-L1, programmed cell death-ligand 1; RT, radiation therapy.

Table S7. Overall survival at 2 and 4 years, PD-L1+ cohort

	No. of Patients	Cumulative Deaths by 2 Years	Probability of OS at 2 Years % (95% CI)	Cumulative Deaths by 4 Years	Probability of OS at 4 Years % (95% CI)
Overall	317	50	81 (77–86)	66	65 (57–75)
Disease Stage at Diagnosis					
IB	112	9	91 (86–97)	11	86 (78–95)
II	126	22	78 (70–87)	31	50 (34–74)
IIIA	79	19	72 (62–84)	24	58 (45–74)
II + IIIA	205	41	76 (69–83)	55	53 (41–69)
Result of Surgery					
R0	304	47	82 (77–87)	62	66 (58–76)
R1	11	2	76 (51–100)	3	38 (9–100)
R2	2	1	NE (NE–NE)[1]	1	NE (NE–NE)[a]
R1 + R2	13	3	63 (36–100)	4	32 (7–100)
Treatment Type					
Resection alone	186	28	82 (76–88)	33	74 (66–84)
Resection + adj. therapy[2]	131	22	81 (74–88)	33	56 (42–75)
Adj. therapy complete	86	11	86 (78–94)	20	58 (42–80)
Adj. therapy incomplete[3]	38	8	74 (59–93)	10	59 (40–87)
PD-L1 Expression					
1-49%	180	24	85 (79–91)	33	67 (54–82)
≥50%	137	26	77 (70–86)	33	64 (54–77)
*EGFR*m Status					
Positive	36	5	85 (74–98)	9	62 (44–88)
Negative	185	23	85 (80–91)	31	68 (55–83)
Unknown	96	22	73 (63–83)	26	63 (52–77)
*EGFR*m Type					
Common	23	3	85 (71–100)	5	63 (40–100)
Uncommon	13	2	84 (66–100)	3	70 (45–100)

[1] Not estimable due to an insufficient number of events.
[2] Systemic therapy and/or RT.
[3] Adjuvant therapy was not completed; explanation unavailable. 7/131 patients receiving adjuvant therapy could not be classified as complete or incomplete.
Adj, adjuvant; CI, confidence interval; *EGFR*m, epidermal growth factor receptor mutation; NE, not estimable; PD-L1, programmed cell death-ligand 1; OS, overall survival; RT, radiation therapy.

Table S8. Disease-free survival at 2 and 4 years, PD-L1+ cohort

	**No. of Patients**	**Cumulative DFS events by 2 Years**	**Probability of DFS at 2 Years % (95% CI)**	**Cumulative DFS events by 4 Years**	**Probability of DFS at 4 Years % (95% CI)**
Overall	317	108	61 (55–67)	125	44 (36–54)
Disease Stage at Diagnosis					
IB	112	16	84 (77–92)	23	68 (55–83)
II	126	48	53 (44–64)	53	40 (29–56)
IIIA	79	44	38 (28–52)	49	17 (6–44)
II + IIIA	205	92	47 (40–55)	102	31 (22–43)
Result of Surgery					
R0	304	102	61 (55–67)	118	45 (37–55)
R1	11	5	50 (26–95)	6	25 (5–100)
R2	2	1	50 (13–100)	1	50 (13–100)
R1 + R2	13	6	48 (26–91)	7	24 (5–100)
Treatment Type					
Resection alone	186	51	67 (60–75)	58	57 (48–68)
Resection + adj. therapy[1]	131	57	52 (44–62)	67	31 (20–47)
Adj. therapy complete	86	35	56 (46–68)	43	34 (23–52)
Adj. therapy incomplete[2]	38	19	46 (31–67)	21	20 (5–87)
PD-L1 Expression					
1-49%	180	57	64 (57–72)	70	45 (35–57)
≥50%	137	51	56 (47–66)	55	43 (30–61)
*EGFR*m Status					
Positive	36	15	55 (40–75)	18	40 (25–65)
Negative	185	55	66 (59–74)	67	43 (31–59)
Unknown	96	38	53 (42–65)	40	46 (34–62)
*EGFR*m Type					
Common	23	10	46 (27–77)	11	37 (19–73)
Uncommon	13	4	69 (48–99)	6	47 (25–90)

[1] Systemic therapy and/or RT.

[2] Adjuvant therapy could not be completed; explanation not captured. 7/131 patients receiving adjuvant therapy could not be classified as complete or incomplete.

Adj, adjuvant; CI, confidence interval; DFS, disease-free survival; *EGFR*m, epidermal growth factor receptor mutation; PD-L1, programmed cell death-ligand 1; RT, radiation therapy.

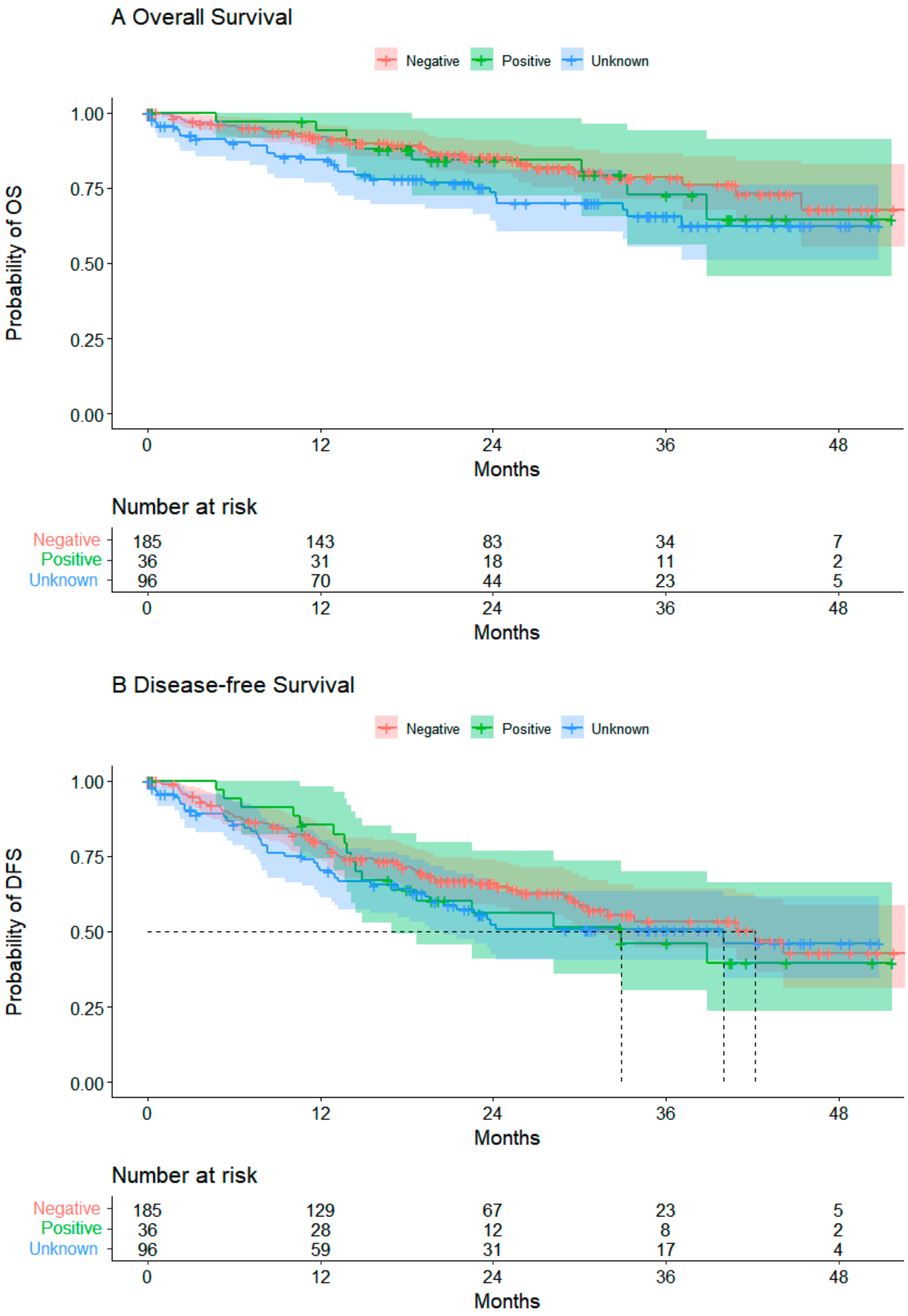

Figure S1. Overall survival (**A**) and DFS (**B**) among PD-L1+ patients stratified by *EGFR*m status. Shaded areas represent 95% CIs. In both comparisons, there was no statistically significant difference in the time to death between patients stratified by negative, positive, and unknown *EGFR*m status (log-rank p-value: > 0.05).

CI, confidence interval; DFS, disease-free survival; *EGFR*m, epidermal growth factor receptor mutation; PD-L1, programmed cell death-ligand 1.
